# Involvement of NO/cGMP Signaling Pathway, Ca^2+^ and K^+^ Channels on Spasmolytic Effect of Everlasting Flower Polyphenolic Extract (*Helichrysum stoechas* (L.) Moench)

**DOI:** 10.3390/ijms232214422

**Published:** 2022-11-20

**Authors:** Marta Sofía Valero, Víctor López, Marta Castro, Carlota Gómez-Rincón, María Pilar Arruebo, Francisco Les, Miguel Ángel Plaza

**Affiliations:** 1 Departamento de Farmacología, Fisiología y Medicina Legal y Forense, Universidad de Zaragoza, 50009 Zaragoza, Spain; 2 Instituto Agroalimentario de Aragón, IA2, Universidad de Zaragoza-CITA, 50830 Zaragoza, Spain; 3 Facultad de Ciencias de la Salud, Universidad San Jorge, Villanueva de Gállego, 50830 Zaragoza, Spain; 4 Instituto de Investigación Sanitaria Aragón (IIS Aragón), 50009 Zaragoza, Spain

**Keywords:** antioxidant, antispasmodic activity, ion channels, gastrointestinal diseases, medicinal plants, polyphenols

## Abstract

Functional gastrointestinal diseases (FGID) are worldwide prevalent conditions. Pharmacological treatments can be ineffective, leading the population to turn to herbal or traditional remedies. *Helichrysum stoechas* (L.) Moench is a medicinal plant traditionally used in the Iberian Peninsula to treat digestive disorders, but its effects on gastrointestinal motility have not been scientifically demonstrated. The aim of this work was to evaluate the antispasmodic effect of a polyphenolic extract of *H. stoechas* (HSM), its mechanism of action and its antioxidant activity. Isometric myography studies were performed in rat ileum, and malondialdehyde (MDA) and 4-hydroxyalkenals (4-HDA) levels were measured in rat jejunum. HSM reduced the integrated mechanical activity of spontaneous contractions. In Ca^2+^-free medium, HSM reduced the concentration–response curve of CaCl_2_ similarly to verapamil. Pre-incubation with the extract blocked the contraction induced by Bay K8644, KCl and carbachol. L-NAME, ODQ, Rp-8-Br-PET-cGMPS, KT-5823, apamin, TRAM-34 and charybdotoxin reduced the relaxant effect of the extract on spontaneous contractions. MDA+4-HDA levels in LPS-treated tissue were reduced by the extract, showing antioxidant activity. In conclusion, HSM showed antispasmodic activity through inhibition of Ca^2+^ influx, activation of the NO/PKG/cGMP pathway and opening of Ca^2+^-activated K^+^ channels. The results suggest that *H. stoechas* could help in the prevention or treatment of FGIDs.

## 1. Introduction

Digestive disorders with symptoms without a clear etiology, as abdominal pain, motility alteration or nausea, are included as functional gastrointestinal diseases (FGID), affecting a large part of the population [1,2]. Irritable bowel syndrome, functional constipation and functional abdominal pain, among others, are included as FGID, which pathophysiology is characterized by alterations in the gut microbiota, mucosal immune function, visceral hypersensitivity and dysmotility [1,3]. In a large-scale multinational study, it was described that the worldwide prevalence of FGID is approximately 40% [4]. The treatment of these pathologies can be complex and sometimes ineffective, and that is why a large part of the population turns to complementary and alternative medicine (CAM), especially herbal medicine, to complement or replace conventional treatment [2,5].

CAM is all practices and products that are not considered part of modern medicine. CAM includes not only herbs and other plant treatments but also non-botanical supplements and mind–body therapies [6]. According to the World Health Organization (WHO) strategy 2014–2023, more than 100 million Europeans currently use CAM, with a much higher number of users in other continents such as Africa, Asia, Australia and North America [7]. CAMs have been and are currently used for the treatment of a wide variety of syndromes [6].

In the FGID treatment, phytotherapy plays an important therapeutic role [2,8,9]. Different online compendia of scientific bodies on medicinal plants as Committee on Herbal Medicinal Products (HMPC) or European Medicines Agency (EMA) reported, in 2017, a total number of 141 medicinal plants, of which 34% are used for the treatment of gastrointestinal diseases [8]. In this sense, the herbal tea form of *Helichrysum arenarium*, for instance, has been approved by the WHO and the EMA for the treatment of digestive problems such as fullness and bloating [10].

The genus *Helichrysum* (Asteraceae), from the Greek “helios” (ἥλιος, sun) and “chrysos” (χρῡσός, gold) by the intense yellow color of its flowers, has approximately 600 species, which has been widely used in traditional medicine throughout the world [11]. *Helichrysum stoechas* (L.) Moench is one of the least known species of its genus. This species, also known as everlasting flower, is distributed in the Iberian Peninsula where it has cooking and culinary uses in beverages, dishes or desserts [12]. The infusion or decoction of its flowers have been traditionally used over the years to improve some problems of the gastrointestinal (e.g., fullness, floating, hepatic and pancreatic disorders), cardiovascular (e.g., hypertension) and respiratory systems (e.g., influenza, flu, common cold), or as a diuretic, suggesting a possible role of this plant for preventing and treating urolithiasis [11,13,14,15,16]. This wide range of therapeutic applications may be due to its anti-inflammatory, antioxidant, antidiabetic, anti-tyrosinase, anti-acetylcholinesterase and antimicrobial activity demonstrated in vitro [17,18,19,20,21,22,23]. To our knowledge, it is only a clinical trial that used *H. stoechas* in combination with other plants in syrup to treat persisting cough in children, showing a reduction in the severity and duration of this symptom [24]. *H. stoechas* has also shown a neuroprotective effect in both in vitro and in vivo assays [18,25] and analgesic effect in vivo [26]. It also has anti-tumorinegic actions as suggested by its inhibition of the proliferation of HeLa cells [18]. Recently, it has been shown to have a hypotensive effect by inducing vascular smooth muscle relaxation through endothelium-independent and endothelium-dependent mechanisms [27].

Reported phytochemicals of different extracts of *H. stoechas* have demonstrated that this species is rich in polyphenols, such as flavonoids and phenolic acids [20,21,22]. In the same way, the phytochemical analysis of this methanolic extract of *H. stoechas*, by the Folin–Ciocalteu method, presented a high content of polyphenols with a strong antioxidant capacity. Furthermore, the main compounds were isolated and identified by chromatography and NMR [18]. These bioactive compounds could explain its range of biological activities. Moreover, various studies reported the potential beneficial effects of polyphenols or their metabolites in several gastrointestinal diseases as inflammatory bowel disease, colitis-associated cancer or FGID [28,29,30,31], although more research is needed in this field. Despite the knowledge about *H. stoechas* is increasing and its flowers are still taken as infusions to treat stomach and intestinal diseases, there is no scientific evidence of the effect of *H. stoechas* on digestive tract. Therefore, the aim of this study was to evaluate the possible intestinal antispasmodic effect of a methanolic extract of *H. stoechas* and its mechanism of action.

## 2. Results and Discussion

### 2.1. Effect of H. stoechas Extract on Spontaneous Contractions

**Figure 1:**
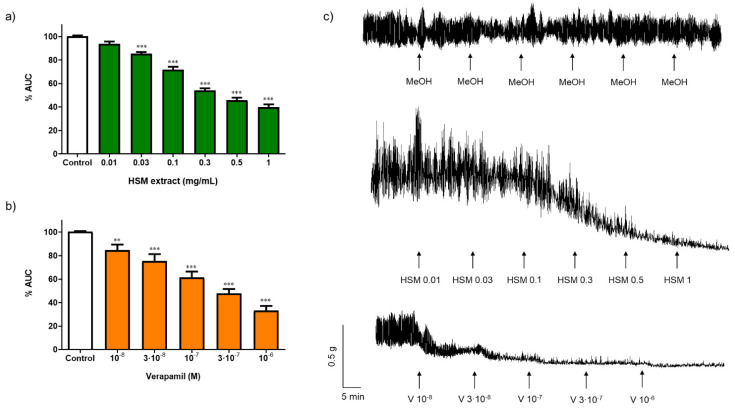
Effect of different concentrations of (**a**) *H. stoechas* extract (HSM) and (**b**) verapamil (V) on the spontaneous contractions in segments of the longitudinal smooth muscle of rat ileum. Data points are mean ± SEM (n = 6–8). ** *p* < 0.01 and *** *p* < 0.001 vs. control (basal spontaneous contractions). (**c**) Representative recordings of spontaneous contractions from cumulative concentration response curves of methanol (MeOH), HSM (0.01–3 mg/mL) and V (10^−8^–10^−6^ M). AUC: area under the curve.

Figure 1a,c show the effect of *H. stoechas* (0.01–3 mg/mL) on the spontaneous contractions in the longitudinal smooth muscle of rat ileum. The *H. stoechas* extract reduced the area under the curve (AUC) in a concentration-dependent manner, with an EC_50_ value of 0.33 mg/mL (0.39–0.28, 95% CI). The amplitude of spontaneous contractions, but not the frequency, was significantly reduced (Table 1). Although methanol was removed from the extract before experiments, the solvent of *H. stoechas*, did not modify the AUC, the amplitude or the frequency of spontaneous contractions of the ileum (Figure 1c and Table 2).

The result reports for the first time the antispasmodic activity of a methanolic extract of *H. stoechas*. Extracts from other *Helichrysum* species also exert antispasmodic activity by reducing the spontaneous motility in intestinal smooth muscle strips. Thus, extracts of *H. plicatum* [32], *H. arenarium* [33,34] and *H. italicum* [35] inhibit the spontaneous contractions in rat ileum, rat and rabbit intestine and mouse ileum, respectively. Furthermore, other plants belonging to the Asteraceae family, such as *Jasonia glutinosa* [36] and *Chrysactinia mexicana* [37], decrease the amplitude of spontaneous contractions without modifying their frequency in the rat duodenum and rabbit ileum, respectively.

The spontaneous rhythmic contractions of the intestinal smooth muscle are necessary for the maintenance of the physiological functions of the intestine. These contractions, initiated in interstitial cells of Cajal, are due to changes in the membrane potential. Ca^2+^ is responsible for gastrointestinal motility. Thus, it has an important role in smooth muscle depolarization and repolarization participating in maintaining the tone, amplitude and frequency of spontaneous contractions and contractile response. The increase in intracellular Ca^2+^ may be due to its entry from the extracellular medium and/or the release of Ca^2+^ from intracellular stores [38].

In our study, verapamil, an antagonist of L-type Ca^2+^ channels, used as positive control, reduced the AUC in a concentration-dependent manner, with an EC_50_ value of 0.17 µM (0.12–0.25, 95% CI). Verapamil also decreased the amplitude, but it did not modify the frequency on spontaneous contractions (Figure 1b,c and Table 1). The decrease in the spontaneous motility induced by the *H. stoechas* extract in the study was similar to that induced by verapamil, an agent that prevents the entry of Ca^2+^ into the cell by blocking the voltage-dependent L-type Ca^2+^ channels (VDCCs), suggesting that *H. stoechas* extract could be acting through the same pathway.

### 2.2. Effect of H. stoechas Extract on Influx of Ca^2+^

**Figure 2:**
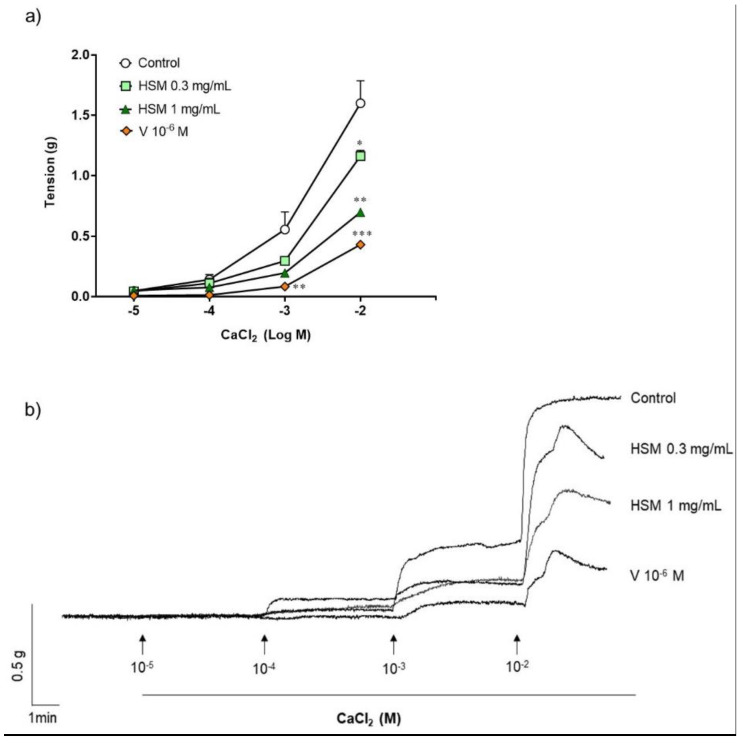
(**a**) Effect of *H. stoechas* extract (HSM, 0.3 or 1 mg/mL) and verapamil (V, 10^−6^ M) on the contraction produced by CaCl_2_ in rat ileum. (**b**) Representative recordings of spontaneous contractions from cumulative concentration of CaCl_2_ in strips pre-incubated with methanol (control), *H. stoechas* or verapamil. Data points are mean ± SEM (n = 6). * *p* < 0.05, ** *p* < 0.01 and *** *p* < 0.001 vs. control.

To examine the effect of *H. stoechas* extract on influx of extracellular Ca^2+^, ileum segments were pre-incubated in a Ca^2+^-free K^+^-rich buffer with methanol (control), *H. stoechas* (0.3 or 1 mg/mL) or verapamil (10^−6^ M). After the incubation period, a cumulative Ca^2+^ curve was performed by adding increasing concentrations of CaCl_2_ (10^−5^–10^−2^ M). As shown in Figure 2, *H. stoechas* shifted the CaCl_2_ response curve down and to the right. *H. stoechas* at 0.3 and 1 mg/mL reduced the maximum response to CaCl_2_ by 27.3% and 56.5%, respectively. Verapamil 10^−6^ M produced a similar relaxation of 73.1% (Figure 2).

### 2.3. Effect of H. stoechas Extract on the Contractions Induced by an Agonist of L-type Ca^2+^ Channels

**Figure 3:**
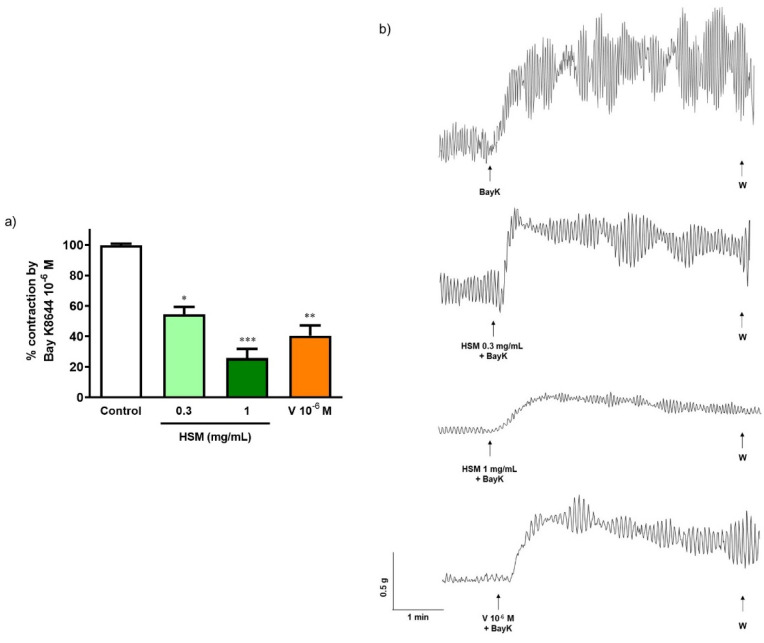
(**a**) Effect of pre-incubation with *H. stoechas* extract (HSM, 0.3 and 1 mg/mL) and verapamil (V, 10^−6^ M) on the contractile response produced by Bay K8644 (10^−6^ M) on rat ileum segments. (**b**) Representative recordings of contractions produced by Bay K8644 alone or in the presence of HSM or verapamil. Data points are mean ± SEM (n = 6–8). * *p* < 0.05, ** *p* < 0.01 and *** *p* < 0.001 vs. control (Bay K8644 alone).

Bay K8644 (10^−6^ M), an agonist of L-type Ca^2+^ channels, produced a contractile response on ileum (control). Pre-incubation with *H. stoechas* extract to 0.3 and 1 mg/mL reduced the Bay K8644-induced contractile response by 45.4% and 74.2%, respectively (Figure 3). Similar pre-incubation with the antagonist of L-type Ca^2+^ channels, verapamil, also induced an inhibition of 59.4% (Figure 3).

These results suggest that *H. stoechas* extract reduces rat spontaneous intestinal contractions by inhibiting Ca^2+^ influx into smooth muscle through the blockade of L-type Ca^2+^ channels.

### 2.4. Effect of H. stoechas Extract on the Contractions Induced by Other Contractile Agents

**Figure 4:**
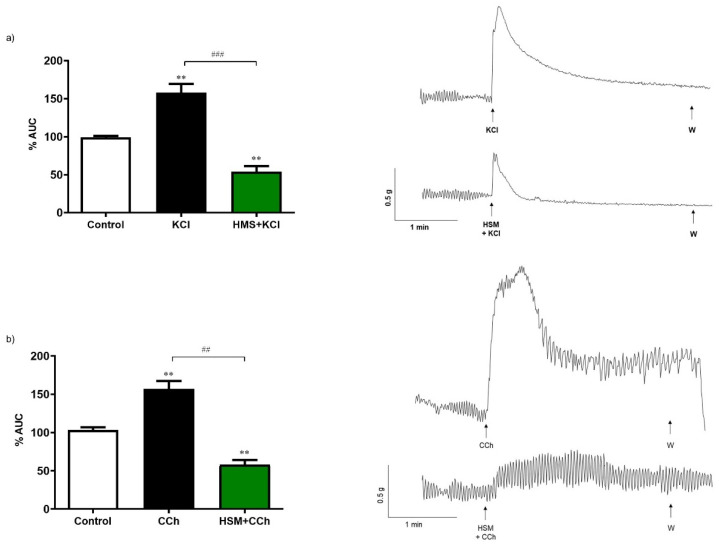
Contractile effect of (**a**) KCl (80 mM) and (**b**) carbachol (CCh, 10^−6^ M) on spontaneous contractions and effect of pre-incubation of *H. stoechas* extract (HSM, 1 mg/mL) on KCl or CCh-evoked contractile responses. Their representative recordings are showed on the right. Data points are mean ± SEM (n = 6). ** *p* < 0.01 vs. control (basal spontaneous contractions) and ^##^ *p* < 0.01, ^###^ *p* < 0.001 vs. KCl or CCh. AUC: area under the curve.

To assess the effect of *H. stoechas* extract on the response to classical intestinal smooth muscle contractile agents, either KCl (80 mM), a membrane depolarizing agent, or carbachol (10^−6^ M), a muscarinic cholinergic agonist, were added to the bath. Pre-incubation of the segments of longitudinal smooth muscle of the ileum for 20 min with the *H. stoechas* extract (1 mg/mL) significantly reduced the contractile response induced by KCl and carbachol by 63.0% and 65.6%, respectively (Figure 4).

KCl at high concentration can evoke a depolarization in the smooth muscle membrane and therefore produce the entry of extracellular Ca^2+^ through the opening of VDCC without any receptor stimulation. In contrast, the muscarinic agonist carbachol induces contraction through the activation of muscarinic cholinergic receptors. These receptors increase the influx of extracellular Ca^2+^ through of activation the non-selective cation channels (ROCCs) as well as the Ca^2+^ output from the sarcoplasmic reticulum through the opening of IP_3_-sentitive Ca^2+^ channels and ryanodine receptors [39]. These results suggest that, in addition to inhibiting extracellular Ca^2+^ influx through VDCC, the *H. stoechas* extract could also act by blocking ROCC and Ca^2+^ release from the sarcoplasmic reticulum.

Extracts from several plants of the Asteraceae family also reduce contractions induced by cholinergic agonists or high K^+^ concentrations, such as *H. italicum* in mouse ileum [35], *H. plicatum* in rat ileum [32], *J. glutinosa* in rat duodenum [36], *Artemisia vulgaris* in rabbit jejunum [40], *Achillea millefolium* in rat ileum [41] and *Chrysactinia mexicana* in rabbit ileum [37]. The results of these works show that, like *H. stoechas*, polyphenolic extracts of these plants inhibit both spontaneous and induced contractions by blocking the entry of Ca^2+^ ions into the smooth muscle cell, mainly through L-type Ca^2+^ channels.

### 2.5. Role of NO and cGMP on the Effect of H. stoechas Extract on Spontaneous Contractions

**Figure 5:**
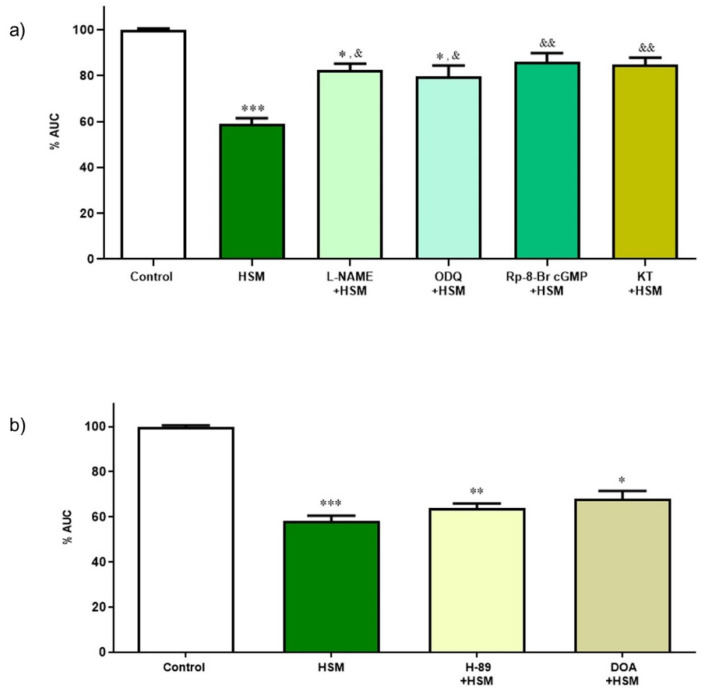
(**a**) Effect of inhibitors of the NO/cGMP/PKG pathway on the relaxant response induced by *H. stoechas* extract (HSM, 1 mg/mL) on the longitudinal smooth muscle of the rat ileum. (**b**) Effect of inhibitors of the cAMP/PKA pathway on the relaxant response induced by the HSM extract on the longitudinal smooth muscle of the rat ileum. Data points are mean ± SEM (n = 6–8). * *p* < 0.05, ** *p* < 0.01, *** *p* < 0.001 vs. control (basal spontaneous contractions) and ^&^ *p* < 0.05, ^&&^ *p* < 0.01, vs. HSM. AUC: area under the curve.

To study whether NO and cGMP were involved in the relaxant response of *H. stoechas* extract, the ileum segments were pre-incubated 20 min before the addition of the *H. stoechas* extract (1 mg/mL) with different substances such as L-NAME (10 µM), an inhibitor of NOS; ODQ (1 µM), a potent and selective inhibitor of soluble guanylyl cyclase; Rp-8-Br-PET-cGMPs (1 µM), an inhibitor of cGKI; and cGKII, KT-5823 (1 µM), a selective inhibitor of PKG. The incubation of all substances reduced the *H. stoechas* extract effect on the AUC of spontaneous contractions (Figure 5a). Inhibitors of the NO/PKG/cGMP pathway had no effect per se on spontaneous motility.

### 2.6. Role of cAMP on the Effect of H. stoechas Extract on Spontaneous Contractions

Like the previous assay, the effect of cAMP was investigated for the relaxant response produced by the *H. stoechas* extract (1 mg/mL) after pre-incubating the ileum segments with H-89 (1 µM), an inhibitor of the PKA, and 2,5-dideoxiadenosina (DOA, 1 mM), an inhibitor of adenylate cyclase. These substances had no effect per se on the spontaneous motility.

None of the substances tested modified the effect of *H. stoechas* extract on AUC of spontaneous contractions of the ileum muscle (H-89 + HSM vs. HSM, *p* = 0.198 and DOA + HSM vs. HSM, *p* = 0.129) (Figure 5b).

These results suggest that the spasmolytic effect evoked by the extract on the longitudinal smooth muscle of the rat ileum is mediated in part by the NO/PKG/cGMP pathway, but not by the PKA/cAMP one.

Under physiological conditions, NO has a very important role in the motility of the GI tract. Thus, an alteration in NO homeostasis produces gastrointestinal motor dysfunction [42,43,44]. NO is a non-adrenergic non-cholinergic neurotransmitter produced by NO synthases and it regulates the intestinal motility through an inhibitory action, relaxing smooth muscle through the intracellular messenger cGMP and PKG [42]. In the smooth muscle, PKG is able to inhibit the Ca^2+^ channels of the sarcoplasmic reticulum, to stimulate the closure of L-type Ca^2+^ channels, to promote the opening of K^+^ channels, to inhibit the RhoA factor and to activate the myosin light chain phosphatase. All of these actions lead to the reduction in cytosolic Ca^2+^, and to dephosphorylate the myosin light chain, thus converging in the relaxation of the smooth muscle cell [45]. In contrast to these results, spasmolytic activities evoked by *H. italicum* extracts in isolated mouse ileum [35] or by essential oil of *Chrysactinia mexicana* in rabbit ileum [37] are not mediated through the NO/cGMP pathway, whereas cAMP is involved in the effects induced by *Chrysactinia mexicana*. This difference may be due to their different phytochemical composition.

### 2.7. Role of K^+^ Channels on Response of H. stoechas Extract on Spontaneous Contractions

**Figure 6:**
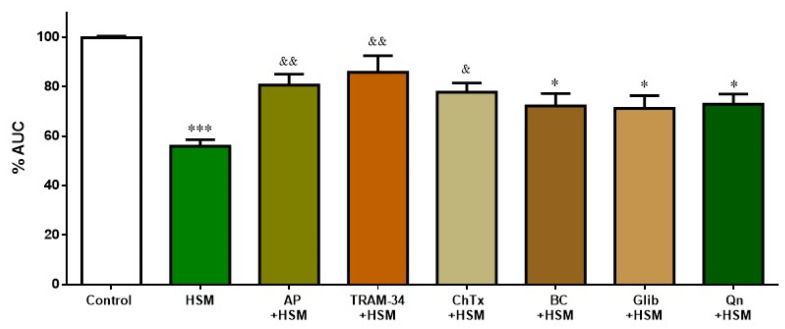
Effect of K^+^ channel inhibitors on the inhibition induced by *H. stoechas* extract (HSM, 1 mg/mL) on the longitudinal smooth muscle of rat ileum: apamin (AP), TRAM-34, charybdotoxin (ChTx), BaCl_2_ (BC), glibenclamide (Glib) and quinine (Qn). Data points are mean ± SEM (n = 6–8). * *p* < 0.05, *** *p* < 0.001 vs. control (basal spontaneous contractions), ^&^ *p* < 0.05 and ^&&^ *p* < 0.01 vs. HSM. AUC: area under the curve.

Figure 6 shows the participation of K^+^ channels in the relaxing response evoked by *H. stoechas* extract (1 mg/mL) after pre-incubation of the ileum segments with various inhibitors of K^+^ channels as apamin, a selective small-conductance Ca^2+^- and voltage-activated K^+^ channel (SKCa) inhibitor (AP, 1 µM); TRAM-34 (1-[(2-chlorophenyl) diphenylmethyl]-1H-pyrazole, a selective inhibitor of intermediate conductance Ca^2+^-activated K^+^ channels (IKCa) (1 µM); charybdotoxin, a specific inhibitor of intermediate- and large-conductance Ca^2+^-activated K^+^ channels (ChTx, 0.01 µM); glibenclamide, an inhibitor of ATP-sensitive K^+^ channels (K_ATP_) (Glib, 10 µM); BaCl_2_, an inhibitor of the inward rectifier K^+^ channel (K_IR_) (BC, 30 mM); and quinine, an inhibitor of voltage-sensitive K^+^ channels (Qn, 10 µM).

Apamin, TRAM-34 and charybdotoxin significantly reduced the effect of *H. stoechas* extract on the AUC from the ileum spontaneous contractions (by 24.7%, 30% and 21.4%, respectively). However, glibenclamide, BaCl_2_ and quinine, although they reduced this effect slightly (by approximately 15%), did not reach statistical significance (Figure 6). K^+^ channels inhibitors had no effect per se on the spontaneous motility.

K^+^ channels also have an important role in maintaining intestinal homeostasis. The activation of smooth muscle K^+^ channels produces cell hyperpolarization and, as a consequence, the closure of the VDCCs, the decrease in cytosolic Ca^2+^ and, finally, the relaxation of smooth muscle. In this study, the relaxant effect induced by the *H. stoechas* extract was significantly reduced by apamin, TRAM-34 and charybdotoxin, suggesting the role of small-, intermediate- and large-conductance Ca^2+^-activated K^+^ channels (IKCa, SKCa and BKCa), respectively. However, the fact that glibenclamide, BaCl_2_ and quinine did not modify the *H. stoechas* extract-induced relaxation showed that ATP-sensitive, inward rectifier or voltage-sensitive K^+^ channels are not involved. Thus, these findings suggest that the spasmolytic activity of *H. stoechas* is mediated through the opening of Ca^2+^-activated K^+^ channels. Similar results were obtained with essential oil of *Chrysactinia mexicana* in rabbit ileum [37].

### 2.8. Potential Additive Effects on Relaxation Induced by H. stoechas Extract

As the Ca^2+^-activated K^+^ channels can be opened by NO, the possible additive effects on the relaxant response produced by *H. stoechas* extract (1 mg/mL) were studied by the combination of TRAM-34 and apamin, as K^+^ channels inhibitors, and L-NAME, as NO inhibitor.

The combination of TRAM-34 and apamin did not modify the AUC of spontaneous contractions shown by the treatment with these K^+^ channel blockers alone. However, the addition of L-NAME with the K^+^ channel inhibitors showed an additive effect of the NOS inhibitor, reaching to an almost complete blockade of the effect of *H. stoechas* on the AUC from spontaneous contractions (Table 3). This result demonstrates an additive effect of NO release and hyperpolarization.

### 2.9. Role of the Rho-Kinase Pathway on the Effect of H. stoechas Extract on Spontaneous Contractions

After showing that the *H. stoechas* extract has clear impact on Ca^2+^-dependent contraction, it was studied whether the Ca^2+^-independent Rho-kinase pathway was involved in the effect of *H. stoechas* extract (1 mg/mL). Thus, ileum segments were pre-incubated with Y-27632 (10^–6^ M), a Rho-kinase inhibitor, and with the combination Y-27632 + extract.

**Figure 7:**
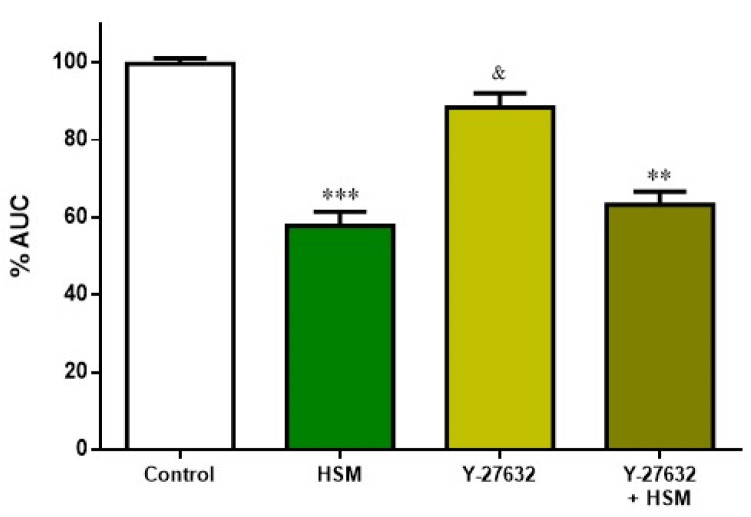
Effect of pre-incubation with *H. stoechas* extract (HSM, 1 mg/mL), Y-27632 (10^−6^ M) and the combination of Y-27632+HSM on the AUC from spontaneous contractions of rat ileum. Data points are mean ± SEM (n = 6–8). ** *p* < 0.01, *** *p* < 0.001 vs. control (basal spontaneous contractions), and ^&^ *p* < 0.05 vs. HSM. AUC: area under the curve.

As in the other assays, the extract significantly reduced spontaneous contractions compared to the control. This effect was not additive when extract was added with Y-27632. Y-27632 per se did not cause a statistically significant change on the AUC from spontaneous contractions of ileum (Figure 7).

The fact that the inhibitor of Rho-kinase Y-27632 did not alter the response of *H. stoechas* extract on spontaneous contractions would indicate that the extract does not change the Ca^2+^ sensitization.

### 2.10. MDA+4-HDA Content

**Figure 8:**
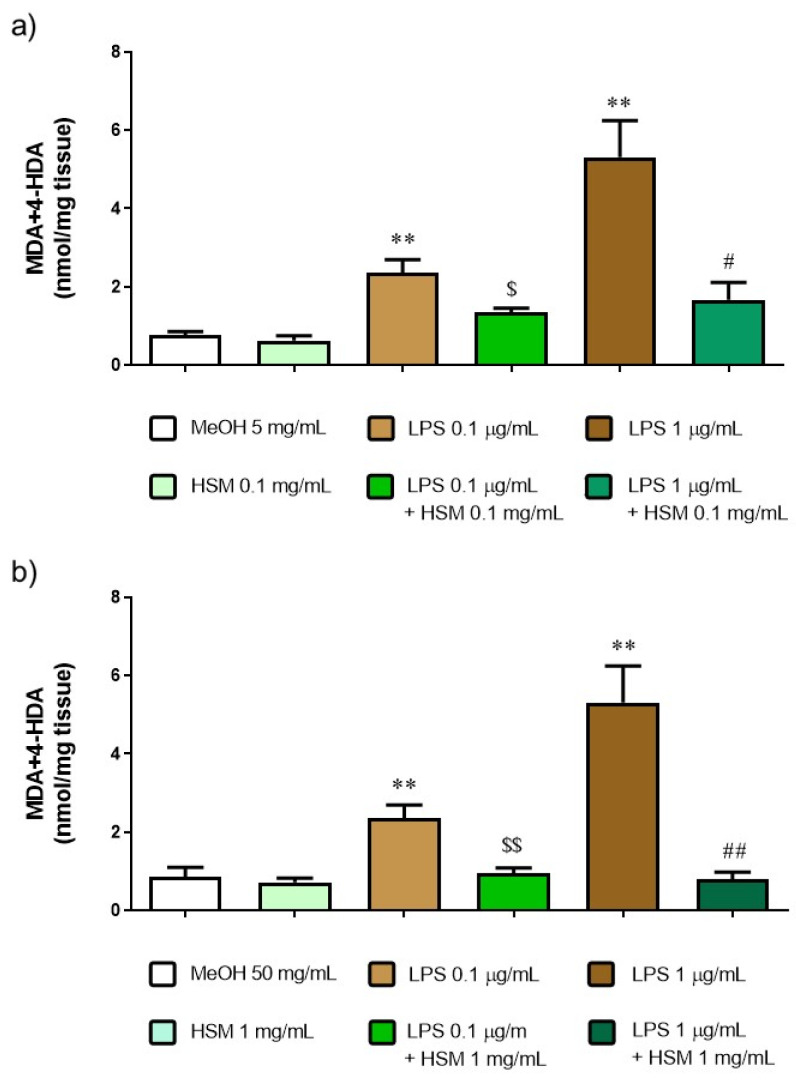
MDA+4-HDA concentration after incubation with MeOH or *H. stoechas* extract (HSM) at either (**a**) low or (**b**) high concentrations, in presence or absence of lipid peroxidation evoked by LPS 0.1 or 1 µg/mL on rat jejunum. Data points are mean ± SEM (n = 5). ** *p* < 0.01 vs. MeOH (control) and HSM, ^$^ *p* < 0.05, ^$$^ *p* < 0.01 vs. LPS 0.1 µg/mL and ^#^ *p* < 0.05, ^##^ *p* < 0.01 vs. LPS 1 µg/mL.

To investigate the role of *H. stoechas* in lipid peroxidation, the content of malondialdehyde (MDA) and 4-hydroxyalkenals (4-HDA) was determined. MDA+4-HDA is one product of polyunsaturated fatty acid peroxidation in the cells and it is produced by an increase in free radicals. Therefore, MDA+4-HDA level is commonly known as a marker of oxidative stress. As shown in Figure 8, *H. stoechas* extract to 0.1 and 1 mg/mL did not increase the level of MDA+4-HDA in tissue relative to MeOH (as control). However, LPS, 0.1 and 1 µg/mL, increased three and eight times the MDA+4-HDA content, respectively. Pre-incubation of *H. stoechas* extract 1 h before the addition of LPS reduced MDA+4-HDA content to a level similar to that of the control (Figure 8).

The NO plays an important role in the regulation of intestinal motility. The formation of reactive oxygen species would produce an alteration in the NO bioavailability, leading to alterations in the intestinal motility [43,44,46,47]. This study showed that *H. stoechas* extract inhibit the content of MDA+4-HDA produced in the tissue treated with LPS, demonstrating the antioxidant activity of the *H. stoechas* extract. Therefore, everlasting flower extract, which has an antioxidant effect, can improve the NO availability and preserve intestinal motor function. Different studies have shown that the administration of flavonoids prevents the structural and functional damage of a digestive tissue through its antioxidant activity by increasing the level of antioxidant enzymes, decreasing lipid peroxidation and modulating the NO level [48,49,50,51,52]. In addition to the phenolic compounds, arzanol, one of the most characteristic molecules of *Helichrysum*, could also explain the reduction in the level of MDA+4-HDA, since it has been shown to have anti-inflammatory and protective effects against lipid oxidation in the plasma membranes of Caco-2 and Vero cells [53].

We find among the spectrum of plants used in FGID those that are characterized by presenting flavonoids [8,54], compounds that have spasmolytic, antioxidant and anti-inflammatory activity, among others. Their inhibitory effects on intestinal motility have been demonstrated in in vitro and in vivo assays [54,55]. Previous studies of the authors showed that *H. stoechas* extracts present a high phenolic profile, with a particular presence of phloroglucinols, being the main compounds arzanol, helipyrone, p-hydroxybenzoic acids, caffeic acids, neochlorogenic acids, 5–7-dihydroxy-3,6,8-trimethoxyflavone, isoquercitrin and quercetagetin-7-O-glucopyranoside [18]. A review shows that the members of the Asteraceae family contain the highest number of antispasmodic compounds, with flavonoids being the group with the highest number of compounds with this activity [56].

Caffeic acid has been shown to have a relaxing effect on different smooth muscles, its most powerful effect being on the ileum. Thus, the spasmolytic effect of caffeic acid was due to the blockade of L-type Ca^2+^ channels and the inhibitory effect on muscarinic receptors [57]. Chlorogenic acid, which is hydrolyzed in the small intestine to caffeic acid, quercetin and other polyphenols, increases NO production by reducing nitrite, and thus produces a relaxing effect on smooth muscle [58]. This result would be in agreement with the relaxation produced by *H. stoechas* extract through the activation of the NO/PKG/cGMP pathway. Additionally, other studies reported that caffeic acid blocks small- and intermediate-conductance Ca^2+^-activated K^+^ channels [59]. These results suggest that caffeic acid may be, at least in part, responsible for the effects evoked by methanolic extract of *H. stoechas*.

Ethanolic extracts of *Drosera madagascariensis* and *Drosera rotundifolia*, rich in isoquercitrin, quercetin and hyperoside, reduce the contractions induced by charbacol and histamine in guinea-pig ileum [60,61]. In the same way, the methanolic extract of *Psidium guajava* showed a spasmolytic effect, which was mainly due to the aglycone quercetin and isoquercetin present in the extract [62]. Thus, the spasmolytic effect of *H. stoechas* extract could be due to some of its constituents, mainly those of phenolic nature.

## 3. Conclusions

The present study reports for the first time the antispasmodic activity of a methanolic extract of *H. stoechas* and its mechanism of action. Thus, the methanolic extract of *H. stoechas* relaxes the smooth muscle of the rat ileum through the reduction in intracellular Ca^2+^ level, the activation of the NO/PKG/cGMP pathway and the opening of Ca^2+^-activated K^+^ channels. The antispasmodic and antioxidant activities of *H. stoechas* extract could help to prevent tissue damage and to preserve intestinal motor function. This study provides new data supporting the traditional use of *H. stoechas* to treat digestive disorders and its use for the development of herbal medicines for the treatment or prevention of FGID.

## 4. Materials and Methods

### 4.1. Reagents, Chemicals and Plant Material

*H. stoechas* aerial parts were collected in July 2014 and a plant voucher was deposited at Universidad San Jorge herbarium (ref. 002-2014). The preparation of the methanolic extract of *H. stoechas* and the identification of its phenolic profile were previously described [18]. The solvent (methanol) was completely removed by rotatory evaporator under vacuum.

Acetylcholine (ACh), carbamoylcholine chloride (carbachol), Bay K8644, apamin (AP), charybdotoxin (ChTx), glibenclamide (Glib), quinine (Q), verapamil (V), H-89 dihydrochloride hydrate, KT-5823, (+)-(R)-trans-4- (1-aminoethyl)-N-(4-pyridyl) cyclohexanecarboxamide dihydrochloride monohydrate (Y-27632), Nω-Nitro-L-arginine methyl ester hydrochloride (L-NAME) and barium chloride dihydrate (BaCl_2_) were obtained from Sigma (Madrid, Spain). TRAM-34, 1H-[1,2,4]oxadiazolo [4,3-α]quinoxalin-1-one (ODQ) and Rp-8-Br-PET-cGMPS were purchased from Tocris (Madrid, Spain). Bay K8644 was dissolved in ethanol. Apamin was diluted in acetic acid. Gliblencamide, TRAM-34 and ODQ were prepared in dimethyl sulfoxide (DMSO). All other chemicals were dissolved in distilled water.

Tissues were incubated in Krebs buffer (in mM: NaCl 120, KCl 4.7, CaCl_2_ 2.4, MgSO_4_ 1.2, NaHCO_3_ 24.5, KH_2_PO_4_ 1, and glucose 5.6), calcium-free Krebs (NaCl 120, KCl 4.7, CaCl_2_ 0, MgSO_4_ 1.2, NaHCO_3_ 24.5, KH_2_PO_4_ 1, glucose 5.6, and ethyleneglycoltetraacetic acid (EGTA) 1) or Ca^2+^-free high K^+^ Krebs ([K^+^]_o_ = 50 mM). The buffers were adjusted to pH 7.4. The compounds of the buffers were obtained from Sigma.

Lipopolysaccharide (LPS) from Escherichia coli O111:B4 was obtained from Sigma.

### 4.2. Animals

Male Wistar rats weighting 200–250 g were purchased from Janvier, LeGenest St. Isle, France. Animals were fed ad libitum with standard feed and free access to water.

The experimental protocols were approved by the Ethics Committee from University of Zaragoza under Project License PI66/17 (18 January 2018). Animal care and use of animals were conducted in accordance with the Spanish Policy for Animal Protection RD 53/2013, RD1386/2018 and RD118/2021, which meets the European Union Directive 2010/63 on the protection of animals used for experimental and other scientific purposes.

### 4.3. Preparation of Ileum Segments

After cervical dislocation, ileum was carefully removed, placed in ice-cold Krebs buffer, and cleaned of fat and adherent connective tissue. The ileum was cut into longitudinal smooth muscle segments (10 mm long) and each segment was individually connected to an isometric force transducer (Pioden UF1, Graham Bell House, Canterbury, UK) for tension measurement. The organ bath, with 5 mL Krebs buffer, was maintained at 37 °C and gassed with 95% O_2_ and 5% CO_2_. Mechanical activity was amplified with a range of 2 mV, recorded and digitalized using a data acquisition system (eDAQ, e-corder 410 (model ED410), Cibertec, Madrid, Spain). An initial tension of 1 g was applied to the preparations to achieve spontaneous contractions. The segments were allowed to equilibrate in Krebs solution for 60 min before use by changing the bath buffer every 20 min.

### 4.4. Experimental Protocols

After the stabilization period, the spontaneous contractions of longitudinal smooth muscle from ileum were recorded. To study the effect of *H. stoechas* extract, methanol (solvent of the extract) or verapamil, increasing concentrations of these compounds were added every 10 min and cumulative concentration–response curves were performed. The spontaneous contractions of the ileum recorded in Krebs solution before agents were considered as the control. The relaxant effect of *H. stoechas* was calculated as the percentage change from the control period (Krebs).

To examine the role of Ca^2+^ influx on the *H. stoechas* extract-evoked relaxation of longitudinal smooth muscle, after incubation with Krebs buffer, the segments were incubated with Ca^2+^-free Krebs solutions for 20 min and then with Ca^2+^-free high-K^+^ buffer. Subsequently, the segments were pre-incubated with methanol, *H. stoechas* extract (0.3 and 1 mg/mL) or verapamil (1 µM) for 15 min and cumulative concentration–response curves for CaCl_2_ (10^−5^–10^−2^ M) were performed by adding each CaCl_2_ concentration every 5 min. The responses to CaCl_2_ obtained in the presence of methanol served as control.

Furthermore, the effect of the *H. stoechas* extract on L-type Ca^2+^ channels was evaluated. Thus, ileum segments were incubated 15 min with *H. stoechas* extract (0.3 and 1 mg/mL) and verapamil (10^−6^ M) before the addition of Bay K8644. The contractile response of Bay K8644 obtained in presence of *H. stoechas* extract or verapamil was compared with the response obtained by Bay K8644 alone (control, 100%).

A similar protocol was used to examine the effects of *H. stoechas* extract (1 mg/mL) on the effects induced by the contractile agents KCl and CCh. The tissue was incubated for 15 min with the extract or the solvent prior to the addition of KCl (80 mM) or CCh (10^−6^ M). Contractions to KCl or CCh in the presence or absence of extract were compared to the control period.

To investigate the participation of AMP/protein kinase A (PKA) and nitric oxide (NO)/GMP/protein kinase G (PKG) pathways on the relaxant response evoked by *H. stoechas* extract (1 mg/mL) in spontaneous motility, segments were incubated 20 min before the extract with L-NAME (10 µM), an inhibitor of NO synthase (NOS); ODQ (1 µM), a potent and selective inhibitor of soluble guanylyl cyclase; Rp-8-Br-PET-cGMPs (1 µM), an inhibitor of cGKI and cGKII; KT-5823 (1 µM), a selective inhibitor of PKG, H-89 (200 nM), an inhibitor of the PKA; and 2,5-dideoxiadenosina (DOA, 1 mM), an inhibitor of the soluble adenylyl cyclase.

To assess whether K^+^ channels were involved in the response of the *H. stoechas* extract (1 mg/mL), 20 min before addition of extract, ileum segments were pre-incubated with apamin, a selective small-conductance Ca^2+^- and voltage-activated K^+^ channel (SKCa) inhibitor (AP, 1 µM); TRAM-34 (1-[(2-chlorophenyl) diphenylmethyl]-1H-pyrazole, a selective inhibitor of intermediate conductance Ca^2+^-activated K^+^ channels (IKCa) (1 µM); charybdotoxin, a specific inhibitor of intermediate- and large-conductance Ca^2+^-activated K^+^ channels (ChTx, 0.01 µM); glibenclamide, an inhibitor of ATP-sensitive K^+^ channel (KATP) (Glib, 10 µM) and BaCl_2_, an inhibitor of the inward rectifier K^+^ channel (KIR), (BC, 30 mM); and quinine, an inhibitor of voltage-sensitive K^+^ channels (Qn, 10 µM).

To study the possible simultaneous role of several pathways in the effects of the *H. stoechas* extract (1 mg/mL), two combinations of inhibitors were also incubated: TRAM-34+AP and L-NAME+TRAM-34+AP, and their effects on the *H. stoechas*-induced relaxation compared with those evoked by each inhibitor alone.

Using the same protocol, the role of the Ca^2+^-independent Rho-kinase pathway on *H. stoechas* extract (1 mg/mL) effect was studied with the Rho-kinase inhibitor Y-27632 (10^–6^ M). In this series of experiments, the response to each substance in the presence of *H. stoechas* extract was compared with the response obtained with the spontaneous contractions (control).

Segments that did not show spontaneous activity were discarded. Thus, each segment served as its own control. Each experimental protocol was performed on ileum segments of longitudinal smooth muscle from 6–8 animals.

### 4.5. Malondialdehyde (MDA) and 4-Hydroxyalkenals (4-HDA)

The tissue concentrations of MDA and 4-HDA (MDA+4-HDA) were used as an index of lipid peroxidation. After cervical dislocation, jejunum was extracted. Jejunum samples were incubated at room temperature in Krebs buffer divided into the following experimental groups: MeOH (as control), *H. stoechas* extract (0.1 or 1 mg/mL), lipopolysaccharide (LPS, 0.1 or 1 µg/mL) and *H. stoechas*+LPS (each at the two referred concentrations). After 1 h of incubation with *H. stoechas* extract or methanol, LPS or its solvent was added. After 2 h, the pieces of jejunum were frozen in dry ice for MDA+4-HDA analysis. The tissues were homogenized in ice-cold Tris buffer (50 mM, pH 7.4) and centrifuged at 3000× *g* for 10 min at 4 °C. In the assay, MDA+4HDA reacts with N-methyl-2-phenylindole to yield a chromophore with maximal absorbance at 586 nm; 1,1,3,3- tetramethoxypropane was used as a standard. Results were expressed as nmol MDA+4-HDA per milligram of tissue.

### 4.6. Analysis of Data

Amplitude and frequency of spontaneous contractions were calculated as the average of peak-to-peak differences and as the number of contractions per minute, respectively, during a 5 min recording period. The area under the curve (AUC) represents the integrated mechanical activity per second (g/s) and normalized to weight (g) of wet tissue. The AUC was calculated as the AUC during the first 3 min of response to the studied substance (AUC_1_) minus the AUC of the spontaneous contractions 3 min before adding the substance (control) (AUC_0_) (AUC = AUC_1_ − AUC_0_). Data were calculated as the percentage with respect to the mean value of the control period and are expressed as mean ± SEM. Normal distribution of the samples was assessed by the Shapiro–Wilk test. Statistical significance was analyzed using the Kruskal–Wallis test (non-parametric) followed by post hoc Dunn’s test. *p* values < 0.05 were considered statistically significant. The concentration of compound that inhibited 50% of the maximal contraction (EC_50_) was expressed as a geometric mean with 95% confidence intervals (IC) and calculated for the concentration–response curve. Statistical analyses and figures were carried out using GraphPad Prism 6.

## Figures and Tables

**Table 1:** Effect of *H. stoechas* (HSM, mg/mL) extract and verapamil (V, M) on the amplitude and frequency of spontaneous contractions of longitudinal smooth muscle of rat ileum. Data are expressed as the percentage of the amplitude and frequency of spontaneous contractions from those of the control period ± SEM (n = 6–8). *** *p* < 0.001 vs. control (basal spontaneous contractions).

HSM (mg/mL)	Amplitude (%)	Frequency (%)	V (M)	Amplitude (%)	Frequency (%)
Control	100.0 ± 3.1	100.0 ± 1.8	Control	100.0 ± 3.8	100.0 ± 0.4
0.01	100.4 ± 4.5	100.9 ± 2.3	10^−8^	89.8 ± 3.1	99.1 ± 1.8
0.03	86.7 ± 7.2	100.0 ± 2.9	3·10^−8^	81.4 ± 4.7	94.6 ± 3.7
0.1	72.7 ± 5.8 ***	96.5 ± 2.0	10^−7^	66.0 ± 4.7 ***	93.9 ± 4.2
0.3	56.4 ± 5.3 ***	96.4 ± 3.0	3·10^−7^	52.4 ± 4.2 ***	93.0 ± 2.8
0.5	55.1 ± 1.0 ***	89.7 ± 4.7	10^−6^	30.5 ± 2.9 ***	92.1 ± 2.9
1	31.2 ± 1.0 ***	87.2 ± 5.8			

**Table 2:** Effect of methanol (MeOH) on the AUC, amplitude and frequency of spontaneous contractions of longitudinal smooth muscle of rat ileum. Data are expressed as the percentage of the amplitude and frequency of spontaneous contractions from those of the control period ± SEM (n = 4). AUC: area under the curve.

	AUC (%)	Amplitude (%)	Frequency (%)
Control	100.0 ± 1.1	101.0 ± 1.0	100.0 ± 1.0
MeOH _0.000012%_	101.5 ± 1.5	100.6 ± 1.2	104.1 ± 1.1
MeOH _0.000036%_	101.7 ± 2.5	99.6 ± 1.3	104.5 ± 1.0
MeOH _0.00015%_	100.1 ± 1.2	99.4 ± 1.1	103.4 ± 1.7
MeOH _0.00027%_	99.0 ± 2.1	98.3 ± 1.3	99.3 ± 1.2
MeOH _0.00075%_	99.1 ± 1.8	97.4 ± 1.5	99.5± 1.3
MeOH _0.0019%_	95.8 ± 2.7	95.2 ± 1.2	98.2 ± 1.1

**Table 3:** Effect of pre-incubation with L-NAME (10^−5^ M), TRAM-34 (10^−6^ M), apamin (AP, 10^−6^ M), and the combination of TRAM-34+AP and L-NAME+TRAM-34+AP on the reduction induced by *H. stoechas* extract (HSM, 1 mg/mL) on the longitudinal smooth muscle of rat ileum. Data points are mean ± SEM (n = 6–8). *** *p* < 0.001 vs. control (basal spontaneous contractions) and ^&^ *p* < 0.05, ^&&^ *p* < 0.05, ^&&&^ *p* < 0.001 vs. HSM. AUC: area under the curve.

Compound	AUC
Control	100
HSM	58.6 ± 2.4 ***
L-NAME+HSM	82.5 ± 2.0 ^&^
TRAM-34+HSM	85.4 ± 5.6 ^&&^
AP+HSM	80.7 ± 4.4 ^&^
TRAM-34+AP+HSM	83.8 ± 2.0 ^&^
L-NAME+TRAM-34+AP+HSM	93.2 ± 0.9 ^&&&^

## Data Availability

Data are contained within the article.
